# Condemn or Treat? The Influence of Adults’ Stigmatizing Attitudes on Mental Health Service Use for Children

**DOI:** 10.3390/ijerph192315951

**Published:** 2022-11-30

**Authors:** Stephanie Lange, Emily Gossmann, Sophie Hofmann, Jörg M. Fegert

**Affiliations:** 1 Clinic for Child and Adolescent Psychiatry/Psychotherapy, University Hospital of Ulm, Steinhövelstraße 5, 89075 Ulm, Germany; 2 Competence Area Mental Health Prevention in the Competence Network Preventive Medicine Baden-Württemberg, Clinic for Child and Adolescent Psychiatry/Psychotherapy, University Hospital of Ulm, Steinhövelstraße 5, 89075 Ulm, Germany; 3 Leadership Personality Center Ulm (LPCU), University Ulm, Kornhausgasse 9, 89073 Ulm, Germany

**Keywords:** stigma, mental health, children and adolescents, prevention, mental health services, health promotion, health care, heteroscedastic ordered probit model

## Abstract

Stigmatizing attitudes towards mental disorders influence parents’ help-seeking behavior for their child’s mental health problems. As untreated mental disorders can cause morbidity and mortality, such parental attitudes are a serious barrier for public health promotion. Therefore, the help-seeking readiness in a distressed child’s broad social environment is essential. However, the role of stigma was unexplored in this context. This study empirically investigated the influence of adults’ stigmatizing attitudes towards mentally disabled people on their readiness to seek professional help for children’s mental health issues. Data from a representative German sample (N = 1906; 52% female) were collected between July and October 2021. A heteroscedastic ordered probit model was used for estimation. An empirical analysis provides evidence for a significant negative relationship between adults’ stigmatizing attitudes and their readiness to initiate mental health support for children (ß = −0.01; *p* < 0.001). Support acceptance seems to be independent of having children. To tackle stigmatizing attitudes and to promote public health, mental health literacy should be fostered through broad-based approaches. Awareness should be raised that children are also entitled to mental health care, just as they are in other health areas. Policy makers need to promote comprehensive information about mental illnesses and create incentives for acute and preventive service use.

## 1. Introduction

Worldwide, the peak age for the onset of any mental disorder is 14.5 years [1]. According to this study, in more than one third of the individuals, a mental disorder occurred before the age of 14, and almost one half occurred before the age of 18. Furthermore, internationally the prevalence of clinically meaningful mental health problems in children and adolescents ranges from 10 to 20% [2], and most of them persist into adulthood and co-occur with multiple mental illnesses [3,4]. These findings highlight the importance of early detection and the use of professional help. However, there is still a low rate of mental health service use among young people. International studies showed that only approximately 25% of children with behavioral problems were referred to medical services [2]. These prevalences are supported by national surveys from Western English-speaking countries such as the UK, Australia and the USA looking at the use of specialist mental health services in children and adolescents with mental disorders [5,6,7,8]. A representative German study involving 2863 families showed that not even half of the affected children with a diagnosed mental illness (5.5% of the whole sample) and only 28.8% of the children showing mental health problems, as measured by the Strengths and Difficulties Questionnaire (SDQ), received mental health treatment [9]. These findings are concerning, given that a delay or lack of appropriate treatment causes long-term negative consequences lasting into adulthood in terms of the individual trajectory, personal and participation impairments, health inequalities, morbidity, mortality, risk-taking behavior and socioeconomic costs [10,11]. Regarding this, it becomes clear that untreated mental disorders are a serious burden for the promotion of public health [12]. From a research perspective, there is thus a large dark field regarding the (health) data of untreated children whose support is hampered by this lack of data.

To address this research gap, it is important to first identify the rationales for why children are not receiving treatment. When investigating the reasons for this public health issue, the research suggests that parental attitudes towards mental health services still influence help-seeking decisions for their children suffering from serious mental health problems [13,14]. Although stigma, in particular, plays a significant role in the prejudging of mental disorders [15,16,17], the role of parental stigma regarding their decision to initiate mental health service use for their children is under-represented in the literature. In particular, quantitative studies with representative data in nonclinical populations and with standardized measurements are lacking [18,19]. Furthermore, to our knowledge there is no study that assesses the attitudes of the social environment on the readiness to seek help for children’s mental health problems. This contributes to disregarding the concept that authorities or adults other than parents can operate as gatekeepers for children’s service use [20]. To fill this gap, the aim of this study was to empirically investigate the relationship between adults’ stigmatizing attitudes and their willingness to use mental health services for children in a representative German sample.

Considering that children and adolescents are primarily dependent on their parents for their access to mental health services [14], previous research focused on the impact of numerous influencing factors in parental help-seeking behavior [8,13,14,21,22,23,24]. The influencing factors analyzed in the previous literature are presented below.

On the structural level, the determinants of the health system, such as health insurances covering costs or the availability of mental health care services, play an important role [25,26,27]. However, these studies were mostly conducted in the USA, where access to the health system differs from many European countries, e.g., Germany. As previously mentioned, even in Germany the use of services for young people with mental health problems is still low [9]. This is surprising, considering that in Germany the costs for a treatment by a psychotherapist are generally covered by the statutory health insurance [28,29]. Furthermore, in a global comparison, Germany has a good supply of mental health offers and specialists. On average there are 54 mental health specialists for 200,000 people [30]. In contrast, almost half of the world’s population lives in countries where, on average, there is just one mental health specialist for 200,000 people. Moreover, Germany ranks third among European countries that have the most psychiatrists [31]. Despite some gaps in the coverage of mental health services for children and adolescents, e.g., in the regional context, Germany has a comprehensive, differentiated and interdisciplinary care system with regard to assistance and care offers [32].

On an individual level, the literature analyzed the influence of parents’ sociodemographic variables on their willingness to seek professional help for their children.

As females report less stigma-related barriers, it might be assumed that there is a gender effect on supporting children in obtaining mental health services [33,34]. Given that gender differences in service use for mental disorders are generally reported and that women seem to be more likely to seek help for their problems [35,36], this may also be transferable to adult-mediated mental health service use for children.

In addition, parents who are younger and have less education seem to be more likely to seek help for their children [18]. However, there are contradictory results with regard to parental education levels, as Zwaanswijk and colleagues (2005) [24] failed to reveal a direct effect in their structural equation model. In line with this, Ryan and colleagues (2015) [22] concluded that there is no sufficient evidence suggesting that parents’ education level is associated with service use, whereas in the context of preventive mental health services, parents with higher levels of education are more likely to enroll in preventive interventions for their children [37]. In line with this, previous research suggested that people with a higher level of education have greater mental health literacy, which includes the willingness to perform an intended help-seeking behavior, at least for their own mental health problems [38]. To conclude, it might be assumed that this may also apply to mental health service initiation for children in need.

Regarding the impact of household income, it is important to consider whether mental health care is publicly funded. In a systematic review, Ryan and colleagues (2015) [22] indicated contrasting results, because many studies showed no relationship between income and service use, and other data suggesting that low and high family incomes predict the use of services for children and adolescents. In general, a lower socioeconomic status seems to predict more service use among children [23].

In line with our assumption, previous research highlights the social context of the child as an important factor for service use, but good empirical data is lacking [20,23]. In order to better support children in this regard, it is therefore important to further explore the influence of the social environment. The existing studies indicate that the perception and assessment of children’s mental health problems by bystanders, e.g., teachers, general practitioners, family members or neighbors, affect parents’ perceptions of mental health symptoms and service need for their children [20,21,22,24]. Based on this, we want to go a step beyond the previous research and assume that if actual or potential parents do not see a need or are unsure about seeking help for their child’s mental health problems, the social environment of the child becomes even more important in initiating the help-seeking process and in supporting the child by themselves. Even if these gatekeepers have no children by themselves or no minor children anymore, their general readiness to initiate mental health support for children is essential for the mental and public health of the upcoming generation.

Moreover, family determinants, for example, the family status, the parent–child relationship, family functioning or the number of siblings, seem to play an important role in the adults’ help-seeking behavior for children [13,22,24]. Regarding the role of family status, e.g., if the parents are divorced, some studies failed to show a link with the use of mental health services for children and adolescents [39,40]. However, there are contradictory results, as the systematic review by Hoffer and Fröhlich-Gildhoff (2019) [21] found parental separation to be a predictor for service use and the systematic review by Ryan and colleagues (2015) [22] showed that being from a single-parent household increased the likelihood for young people to receive mental health services.

A positive impact on the intention of help-seeking for children was found in parents who personally used mental health services themselves [41]. This finding is supported by Oh and colleagues (2015) [8], who showed that mothers’ personal experiences of seeking professional help for their mental health problems predicted the access to service use for their children. In addition, other individual parental factors, e.g., psychopathological aspects, which means that psychological symptoms are present in the parent or the parent reports having experienced a mental health problem [13,22], and individual knowledge regarding treatment options [14,42,43] are recognized as having a significant effect on the help-seeking behavior for children and adolescents.

Previous research shows that adults’ help-seeking behavior for children might depend on their general attitudes towards mental health services [14]. This could be transferable to attitudes towards health services in general, even in the field of physical care. For example, Bellettiere and colleagues (2017) [44] analyzed a sample of 34,843 parents and showed that children whose parents reported that they do not trust doctors had lower odds of receiving well-child care and influenza vaccinations.

If we regard vaccinations as an example of a preventive measure to protect children from severe diseases, research shows that parents who have good health literacy regarding vaccinations [45] or receive routine vaccinations themselves [46] are also more likely to have their children routinely vaccinated. With regard to the use of other preventive health services, findings also suggest that a mother’s good dental attendance pattern influences the dental health of her 5-year-old child [47]. Furthermore, in comparison with nonattenders, children of parents who had regular dental visits were also more likely to show up for check-up visits (22% vs. 7%), had more preventive services (24% vs. 5%), e.g., fissure sealing, and were not seeing a dentist only for symptomatic reasons [48]. These results indicate that parents’ preventive health care, as measured by doctor visits and having health checks, has an impact on their children’s preventive health care and health service use. Therefore, it may be assumed that this mindset may also be adapted to mental health service use. This assumption can be supported by empirical findings that show that people, regardless of whether or not they have children, who attend annual health check-ups and have better health literacy in general are also more likely to have better mental health literacy [38]. Mental health literacy in this study was measured by intended positive help-seeking behavior, at least for one’s own mental health problems, and the readiness to disclose mental health problems, not only to professionals but also to informal resources. In this regard, it might be assumed that adults who have positive attitudes towards preventive physical as well as mental health care and towards disclosing their own mental health problems are also more likely to initiate an early treatment for children’s mental health problems.

Although research has shown that parental attitudes surrounding mental health and towards mental health services can influence help-seeking decisions [13,14], the role of parental stigmatizing attitudes towards mental illnesses and treatment is still under-represented in the empirical literature. This is surprising, given that there is good evidence that stigma affects mental health care use in general [15,17,49]. Therefore, Hinshaw (2005) [16] consequently described stigmatization as “the central issue […] for the entire mental health field” (Hinshaw, 2005, p. 714) [16]. This is supported by the findings of a meta-analysis and systematic review that reported an association between peoples’ own negative attitudes towards mental health treatment, their stigmatizing attitudes towards people with mental disorders and less active help-seeking [33,50]. These findings suggest that stigma-related beliefs and behaviors also influence the likelihood of parents and adults in general to seek help for affected children. In this context, parents may fear being labeled as inadequate or that their children might be treated differently when obtaining treatment [18]. These assumptions are supported by quantitative data from a parent sample collected from a rural pediatric primary care practice, showing that parental concerns regarding stigma predict parental help-seeking [18] and results from a community sample showed that less intended stigmatizing behavior in parents is associated with increased service use in young people [19].

To the best of our knowledge, there is no study that links adults’ stigmatizing attitudes with their help-seeking behavior for children’s mental health problems with regard to representative population data.

In summary and in line with the existing research, the following hypothesis was derived.

**H:**  *Adults’ stigmatizing attitudes towards people with mental disorders have a negative influence on the mental health service use for children.*

## 2. Materials and Methods

### 2.1. Sample

The hypothesis was tested on a representative German sample collected between July 2021 and October 2021 by the USUMA GmbH company in Berlin. The survey focused on well-being and psychological health. Data collection was divided into two steps. First, in-person interviews guided by a structured questionnaire were conducted. Second, the participants completed an associated questionnaire by themselves.

A three-staged methodology was conducted for sampling. First, a systematic area sampling of geographic units in Germany was processed. Subsequently, households within these geographic areas were selected by means of the random route procedure. Therefore, trained interviewers started at a sample point within each geographic area, and their step width was specified to collect valid addresses randomly. The interviewers had to list all doorbell signs to a certain number and in a specified step width related to a predefined random route walk-in regulation. The aim of this procedure was to ensure a quality-appropriate exhaustion of the household sample. Afterwards, the households selected in this way were contacted and asked to give an interview. Third, a Kish selection grid was applied to randomly identify participants from multiperson households. For the Kish selection grid, initially all members of a household who fit the inclusion criteria were listed in a scheme on the address list. The inclusion criteria for study participants were a minimum age of 16 years and sufficiently mastering the German language. Therefore, all men and women in the target household were entered into this numbered scheme according to their age in a descending order and separated by gender. The target person for the interview was the person whose number was the first in a preprinted order of random numbers. By using the Kish selection grid, the selection of the interview participant occurred independent of the interviewer and the contact person. The sampling initially resulted in the selection of 5934 households, but 0.3% of the selected households could not be included in the survey because they did not have a resident who met the criteria. A further 0.1% could not be considered due to unoccupied habitations. Finally, the response rate was about 42.6%, resulting in a sample size of 2515.

The survey was approved by the Ethics Committee of the Medical Department of the University of Leipzig (298/21-ek) and was conducted in accordance with the Declaration of Helsinki. It met the ethical guidelines of the International Code of Marketing and Social Research Practices of the European Society of Opinion and Marketing Research and the International Chamber of Commerce.

Randomly selected persons were verbally informed about the survey, the voluntary nature of participation and their right to withdraw. Informed consent was obtained from all participants. It included transparently written information about the survey. At least one caregiver was consulted to provide informed consent for legally minor participants. In addition, a written privacy statement was given to all study participants.

### 2.2. Measures

The dependent variable adult help-seeking for children measures the attitude of adults towards mental health treatment initiation for their (potential) children on a five-point scale. Survey participants were asked the following question: “*I would have my child treated psychiatrically or psychotherapeutically if I noticed that he or she was developing mental health problems. If you don*’*t have children answer the question from your gut*” (1= “I totally disagree” and 5 = “I fully agree”).

The independent variable stigmatizing attitudes captures the personal agreement of adults with stereotypes about people with mental illnesses. It was measured by the short form of the Self-Stigma of Mental Illness Scale (SSMIS-SF), which is an approved and validated instrument to capture stigmatization [51]. The variable was operationalized by the following five items on a five-point scale (1 = “I totally disagree” and 5 = “I fully agree”): (1) *Most mentally ill people are unpredictable*, (2) *most mentally ill people will not recover or get better*, (3) *most mentally ill people are dangerous*, (4) *most mentally ill people are unable to take care of themselves* and (5) *most mentally ill people are to blame for their problems*. The scale reliability coefficient (Cronbach’s alpha) for the stigma score in this sample was about 0.89. The variable was operationalized as a sum value from the individual items.

Taking previous research into account, the model controlled for the effects of gender, age, education, income, adults’ history of psychotherapy, the family status, the attitude towards preventive health care activities and the individual readiness to disclose health problems.

Gender was added as a categorical (female, male, or nonbinary), and *age* was added to the model as a continuous control variable. Education was operationalized as a categorical variable with the following graduations according to the German school system: 1 = not specified/other graduation; 2 = pupil of a general education school (included different types of schools, e.g., elementary school, evening classes, preschools or high schools) [52]; 3 = without main school diploma (can be compared to the American junior high school and covers years five to nine, or the last five years of the compulsory nine years at school in Germany) [53]; 4 = main school diploma; 5 = secondary school leaving certificate (equivalent to the GED in the U.S.) [53]; 6 = graduation from a polytechnic high school (the polytechnic high school was the general form of school for all pupils in the former German Democratic Republic and compromised ten classes) [54]; 7 = technical college specialized school degree; 8 = general or subject-linked university entrance qualification (a qualification that is approximately equivalent to the passing of the American SAT exam and is usually taken at the end of the 13th school year in Germany) [53] and 9 = completed university studies or studies at a university of applied sciences. Income was added to this model as a continuous measure. It represented the equivalent income of the surveyed study participants. The model controlled for the adult history of psychotherapy (“*Have you ever been in psychiatric or psychotherapeutic treatment?*”*)*. The operationalization followed a three-point scale capturing the dimensions “No”, “Yes, but currently I am not in any treatment” and “Yes, and I am currently still being treated”. To control for family status, we integrated a control variable with the following three categories: 1 = no children, 2 = child(ren) and married/cohabitating, and 3 = child(ren) and single/divorced/widowed/married and living separately. We integrated a self-developed control variable to our model, which depicted the readiness for preventive health care in four medical areas. The variable preventive health care controlled for the hypothetical and actual use of preventive services in the field of dentistry (“*Do you attend the dental check-ups to which you are entitled (once per calendar half-year)?*”), cancer screening *(*“*Do you take advantage of the screening examinations for the early detection of cancer to which you are entitled?*”), general physical health (“*Do you take advantage of the health checks to which you are entitled (from the age of 35, every three years)?*”*)* and mental health *(*“*Would you attend regular screenings for early detection of mental health problems?*”). All items were recorded on a three-point scale (“yes, regularly”; “yes, I have done before but not regularly” and “no”). The variable preventive health care was derived from the sum of all four items. Furthermore, the individual readiness to disclose health problems was added as a control variable. It controlled for the readiness to disclose both physical/somatic and mental health complaints to one’s social environment, each on a five-point scale (1= “I totally disagree” and 5 = “I fully agree”). The variable was calculated as the sum value of the items (1) “*If you notice that you are developing mental health problems, would you tell your family or friends about it?*” and (2) “*If you notice that you are developing physical/somatic discomfort, would you tell your family or friends about it?*”.

### 2.3. Model and Estimation

As mentioned in the introduction, we assumed that the attitudes of possible gatekeepers in the general population may also function as an enabling or inhibiting factor in initiating mental health treatment for children. The hypothesis was thus tested based on data including adults with and without children.

A descriptive evaluation of the individual variable characteristics is provided by Table 1. Table 2 shows the descriptive statistics and correlations.

As the dependent variable was operationalized by discrete values, where larger values were on the right side related to the real line, an ordered probit model was used for estimation [55]. Ordered probit models are frameworks for the statistical analysis of ordinal survey data and are described by the equation below with the following specifications [55,56]: i indexes the number of observations (reaching its maximum at the sample size, N). y_i_ represents the individual response of participant i. Due to the scale of the dependent variable, it can take integer values between one and five. y_i_^*^ (with –∞ < y_i_^*^ < +∞) represents the underlying latent construct, which operationalizes the propensity of participant i to agree with the item. x_i_ is a vector of variables that explains the attitude of a survey participant (explanatory variables). The parameter vector ß is assumed to contain no intercept [55,56].

$$y_{i}^{*}=x_{i}^{\prime }\beta +u_{i}\;\mathrm{with}\;i=1,\ldots ,N.$$

The individual parameters of the vector were assigned as follows: ß_1_, stigmatizing attitudes; ß_2_, gender; ß_3_, age; ß_4_, education; ß_5_, income; ß_6_, adult history of psychotherapy; ß_7_, preventive health care; ß_8_, family status and ß_9_, readiness to disclose health problems with family and friends. The relationship between y^*^ and y is given by the following equations, with κ representing the cut points of the model [56]:

$$y=1\;if-\infty <y^{*}<\kappa_{1}$$

$$y=2\;if\;\kappa_{1}<y^{*}<\kappa_{2}$$

$$y=3\;if\;\kappa_{2}<y^{*}<\kappa_{3}$$

$$y=4\;if\;\kappa_{3}<y^{*}<\kappa_{4}$$

$$y=5\;if\;\kappa_{4}<y^{*}<\infty$$

As misspecifications of the model lead to inconsistent parameter estimators [55], we tested for heteroscedasticity. The results indicated the presence of heteroscedasticity for both the baseline model (model 1) and the full model (model 2) (for details see Section 3.2). Accordingly, heteroscedastic ordered probit models were used for estimations.

## 3. Results

### 3.1. Descriptive Analysis

Due to missing values, the main sample (all participants) included 1928 adults, with 47.93% male, 52.02% female and 0.05% nonbinary participants with a mean age of 55.36 years. The sociodemographic characteristics of the participants are described in Table 1.

**Figure 1:**
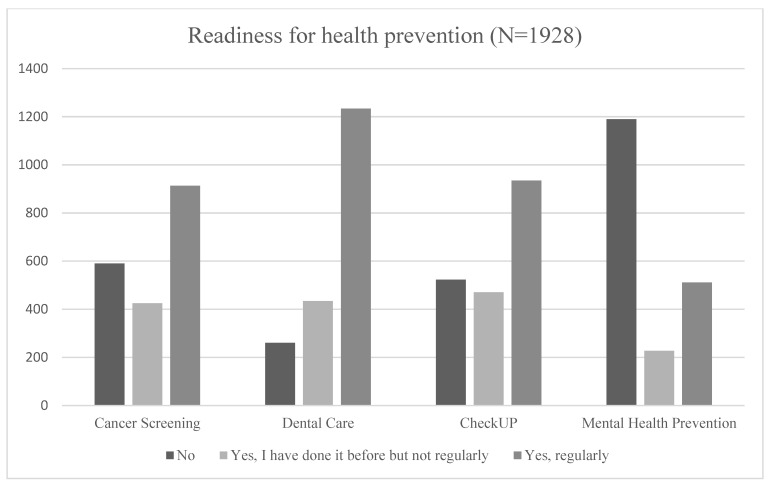
Readiness for health prevention in different medical fields (cancer screening, dental care and check-ups are regular precautionary offers; mental health prevention was asked as a hypothetical offer).

Most of the participants had a main school diploma (29.93%) or a secondary school leaving certificate (31.28%), and they received a mean equivalent income of EUR 2032.63 per month. In total, 62.40% of the adults were parents. Regarding health prevention, the descriptive results showed a differentiated picture (see Figure 1).

Most of the respondents indicated the regular use of preventive dental care (64%), cancer screening (47%), and general health check-ups (48%). However, about 62% would deny seeking mental health care prevention. Only about 27% would be willing to use preventive services in the field of mental health. It should be noted, however, that the question referring to mental health care prevention was hypothetical, as there is currently no such provision in Germany. The data, furthermore, showed that there was a slightly greater readiness to disclose somatic/physical health problems to family and friends compared to the disclosure of mental health problems by the adults themselves. About 85.27% of the participants never had a psychotherapeutic/psychiatric treatment before. Possibly as a consequence of this, we also found at least a medium tendency to stigmatize persons with mental health problems (mean: 10.80; standard deviation: 4.5; min, max [5;25]) (see Table 2). The data showed virtually no difference in stigmatizing behavior between people with children and those without.

**Table 2:** Descriptive statistics and correlations (N = 1906). Notes: * *p* < 0.05; ** *p* < 0.01; *** *p* < 0.001.

Variables	Mean	Standard Deviation	Min	Max	2	3	4	5	6	7	8	9	10
1. Adult help-seeking for children	3.94	1.14	1	5									
2. Stigmatizing attitudes	10.80	4.53	5	25	1.00								
3. Gender	1.52	0.50	1	3	−0.11 ***	1.00							
4. Age	55.36	15.29	16	101	0.09 ***	0.008	1.00						
5. Education	5.58	1.73	1	9	−0.10 ***	−0.05 *	−0.15 ***	1.00					
6. Income	2036.86	1031.08	125	7500	−0.07 **	−0.09 ***	−0.11 ***	0.34 ***	1.00				
7. Adult history of psychotherapy	0.18	0.47	0	2	−0.13 ***	0.11 ***	−0.06 **	−0.02	−0.08 ***	1.00			
8. Preventive health care	4.53	2.54	0	8	−0.15 ***	0.26 ***	0.17 ***	0.05 *	0.12 ***	0.006	1.00		
9. Family status	0.99	0.86	0	2	0.02	0.03	0.12 ***	0.004	0.01	−0.09 ***	0.11 ***	1.00	
10. Readiness to disclose health problems	6.72	2.22	2	10	−0.26 ***	0.12 ***	−0.02	0.04	0.08 ***	0.03	0.29 ***	0.09 ***	1.00

### 3.2. Results from Estimation

The hypothesis predicted a negative relationship between stigmatizing attitudes and adult help-seeking behavior for children. To test for heteroscedasticity, likelihood ratio tests of homogeneity were conducted for the main model (N = 1906). Significant results indicated the presence of heteroscedasticity for both model 1 (baseline model) (χ^2^ = 18.56; *p* < 0.001) and model 2 (full model) (χ^2^ = 89.79; *p* < 0.001). Consequently, a heteroscedastic ordered probit model was used for the empirical estimation. The results are shown in Table 3.

The hypothesized relationship was supported by both model 1 (baseline model with ß_1_ = −0.05; *p* < 0.001) and model 2 (full model with ß_1_ = −0.01 and *p* < 0.001), providing evidence for a negative influence of stigmatizing attitudes on adult help-seeking behavior for children. In line with the existing literature, model 2 also showed a significant impact for the control variables gender (ß_2_ = 0.04; *p* < 0.05), age (ß_3_ = −0.001; *p* < 0.05) and income (ß_5_ = −0.00003; *p* < 0.01). The assumption that a higher willingness to use preventive services for oneself has a positive influence on mental health care service use for children was also confirmed (preventive health care, ß_7_ = 0.02; *p* < 0.001). The adults’ history of psychotherapy indicated a weakly significant impact (ß_6_ = 0.04; *p* < 0.1). Furthermore, the individual readiness to disclose health problems to family and friends had a highly significant influence (ß_9_ = 0.08; *p* < 0.001). In accordance with some of the existing literature, the empirical estimation showed that the family status seemed to have no impact on adult help-seeking for children. Education also had no impact.

## 4. Discussion

The aim of the present study was to empirically investigate the influence of adults’ stigmatizing attitudes on their willingness to initiate mental health support for children, regardless of whether the respondents themselves have children or not.

As hypothesized, empirical estimations with data from a representative German sample provided evidence for a negative relationship at a highly significant level, indicating that a high degree of stigmatization among adults leads to low uptake of mental health services for children in need. In addition, the personal use of preventive services as well as the individual readiness to disclose health problems had a highly significant influence on service initiation for children. To the best of our knowledge, this is the first study analyzing the relationship between adults’ own readiness for prevention and the initiation of preventive mental health activities for children. The general willingness to make provisions was particularly high in the area of dentistry. In contrast, the aversion of adults to use regular preventive health care in the area of mental health was almost as high. This implies that a lot of convincing is still necessary regarding the readiness of adults to engage with mental health prevention and therefore contribute to a better public health.

The influence of further control variables largely corresponded to the results of previous studies. Nevertheless, a particularly important finding of our study remained that family status and education did not appear to influence mental health service utilization for children. Even though former research occasionally also failed to show associations between those variables and caregivers’ help-seeking behavior for their children, as shown in the introduction [22,24,39,40], our model was expected to show different results, as it also considered people without children. One may argue that having children, would make a significant difference in the decision to initiate help for children in need compared to nonparents. Moreover, as we hypothetically asked for help-seeking intentions for childhood mental health problems in a mixed sample, it may have been assumed that education would at least play a significant role one way or the other. This finding is in particular surprising, as income appeared to have a significant negative relationship with adults’ intended help-seeking behavior in our model, meaning that a higher income results in less readiness for children’s mental health service use, and income and education are likely to interact [57]. Accordingly, it is possible that the education variable was biased by the social desirability aspect in our survey. The existing literature showed that social desirability is an important predictor in surveys asking for mental-health-related stigma and intended behavior regarding people with mental health problems [58]. In addition, by asking about the help-seeking behavior for children in need, the social desirability in respondents might be even more distinctive. It may be assumed that social desirability plays such an important role in this relationship that education no longer has a significant impact. However, since we found no study that asked for the hypothetical readiness for mental health service use for children in a mixed sample with parents and nonparents, our findings in terms of education have to be further discussed and tested in other empirical studies.

Readiness, on the other hand, is influenced by sociodemographic data, personal experience with mental health treatment and the individual mindset towards health care.

Our results were in line with the very few studies conducted so far. Nevertheless, the existing research mainly referred to qualitative data [59], and existing quantitative studies showed substantial methodological issues [18]. Moreover, our analysis differed from existing work in that it was based on a representative sample. The existing research mainly analyzed clinical trials [18] or special settings, e.g., urban primary care [60] and rural or community samples [19,27], to find factors influencing the willingness of parents to initiate mental health support for their children. This contains the probability of an important bias, “as it excludes families deterred from seeking help or who did not see mental health service input as useful or necessary” [19]. With the representative sample, we were able to find results for a significantly larger population compared to prior studies [41].

Previous studies further criticized the lack of use of evaluated measurement instruments for the operationalization of stigma [14,18]. In our questionnaire, we used the short form of the Self-Stigma of Mental Illness Scale (SSMIS-SF) to measure the stigmatizing attitudes of adults. We thus contribute to filling this research gap.

Furthermore, our research goes beyond existing studies that mainly focused on parents as gatekeepers for children’s access to mental health services. By using a representative sample, we were also able to integrate bystanders in the children’s environment in the analysis to show that having children does not appear to influence the relationship between stigmatizing attitudes and the readiness to seek help for mentally burdened children. In conclusion, if actual parents or potential future parents are not willing to seek help for their own children, even though it may cause disadvantages for their own child, this highlights the importance of the children’s social environment even more. Therefore, formal (professionals or nonprofessionals from primary health, child welfare, juvenile justice or education) and informal bystanders (family, friends or volunteers) play a major role in identifying mental health problems and initiating mental health support for children. This conclusion is supported by previous research by Gulliver and colleagues (2010) [61], who showed that social support and the encouragement of other individuals in the social environment in particular prove to be a conductive factor in young people’s uptake of mental health support. We thus considered the “gateway provider model” by Stiffman and colleagues (2004) [62] to address the social networks of children. This is an important contribution to emphasize that the help-seeking process should not start with the doctor’s office but much earlier. The impairment must be perceived at an earlier time, and a need for action must be derived. For example, bystander attention might be drawn when severe social impairments occur and they can assist with knowledge about services, consultation and referral or just act as a liaison to services [62].

The importance of this approach becomes particularly clear when considering the high prevalence as well as the early onset of mental health problems described in the introduction. In addition, in the context of the COVID-19 pandemic, the prevalence of mental health problems in children and adolescents increased worldwide compared to prepandemic estimates [63,64]. This is in line with findings from Germany, showing that the prevalence of the psychological burden nearly doubled and that the health-related quality of life decreased significantly [65]. These results emphasize the need for immediate, early and targeted professional help to prevent even more persistent and recurrent psychological disorders in minors and to promote public health in the future.

### 4.1. Limitations

Although this study improved previous research for the aforementioned reasons, some limitations have to be considered. First, we asked adults about their willingness to treat their child psychiatrically or psychotherapeutically if they noticed any mental health problems. This means it is a hypothetical question and does not measure their actual or past behavior. Further studies were already criticized for asking parents hypothetical questions, as the answers “cannot be taken as evidence of actual help-seeking for adolescents in need” [13]. However, as the aim of our study was to also explore the stigmatizing attitudes towards mental disorders of possible gatekeepers and their willingness to seek help for children, hypothetically asking these people without children about their attitude is essential. Furthermore, the “theory of planned behavior” by Ajzen (1991) [66] indicated and empirical studies gave evidence that attitudes are highly linked with actual behavior in the decision to seek help for mental health problems (for an overview see [50,67]). However, it has to be mentioned that our dependent variable adult help-seeking for children was measured by a single item and therefore did not represent a validated measurement instrument. The use of a multi-item scale would capture more variance in the construct of participants’ help-seeking attitudes.

Another important limitation of our study concerns the measurement of the independent variable itself. Even though we used an evaluated measurement instrument to assess respondents’ stigmatizing attitudes towards people with mental disorders, these items gave no information about respondents’ perception about the public stigma towards mental health problems and mental health service use. However, this has repeatedly been shown to be an important factor in whether adults seek personal treatment [15,68]. However, parents and adults in the social environment of the treated child may be concerned that society would blame and label them or treat them differently and not only the mentally burdened child as the primary carrier of the stigma [18]. In conclusion, believing that the parents themselves rather than the child will be the target of public stigma can also make a difference in the decision to initiate professional help for the child [67,69]. In addition, this could possibly be associated with the relationship the respondent has with the child. For example, a close person to the child (e.g., an aunt or brother) may be more afraid of the public stigma.

As mentioned above, the lack of influence of education in our model may be the result of a biased response behavior. Moreover, in recent years and especially in the context of the COVID-19 pandemic, more public awareness of mental health problems was raised and more antistigma and antidiscrimination work towards mental disorders was performed in Germany and globally [70,71,72]. In conclusion, one limitation of our study could be that we did not control for social desirability.

Additionally, we did not control for all of the well-known structural and individual determinants that previous studies focusing on parents had already identified as influencing factors in seeking help for minors, e.g., psychopathological aspects, individual knowledge regarding treatment options, the parent–child relationship, etc. [13,14,22,42,43]. However, we controlled for the adults’ history of psychotherapeutic care, and by doing so we assumed that we implicitly partly captured the individual knowledge concerning mental health aspects and their symptoms. Moreover, the hypothetical readiness for mental health prevention may reflect the existing knowledge in the field of mental health.

Finally, the cross-sectional design of our study precludes causal interpretations. Therefore, the following implications and conclusions drawn from our analysis are limited and only apply to the German population. Furthermore, as the inclusion criteria for interview participants were restricted to people with a permanent residence in Germany with sufficient mastery of the German language, the generalizability of our research results needs to be taken with care with regard to vulnerable populations (e.g., homeless people) and populations with different ethnical backgrounds.

### 4.2. Theoretical and Practical Implications

The findings of our analysis highlight, on the one hand, theoretical and empirical implications for future research and, on the other hand, practical implications for policy makers, service providers and people working in the mental health sector.

From a theoretical and empirical point of view, future research should measure self-stigmatizing attitudes as well as the perception of public stigma with evaluated measurement instruments. Therefore, it is also essential to capture the concern of the respondent who will be the target of the public stigma [18]. Additionally, it is important to assess the quality of the relationship not only of the parents to the child but also the kind of relationship the interviewed bystanders have to the child. To take it a step further, future research could not only ask hypothetical questions about the willingness of respondents to seek psychological help for children but also complement it by asking about actual or past supporting and helping behavior. To add, future research could analyze data from parents of mentally ill children with regard to the reasons for differences in those who seek treatment for their mentally ill child and those who do not. Furthermore, it is important to include a validated measurement scale controlling for social desirability by assessing mental-health-related stigma in respondents and add all of the well-known predictors in parents that the previous literature already identified as having an influence on adults’ help-seeking behavior for children and adolescents. Finally, longitudinal data on the relationship between adults’ stigmatizing attitudes and their willingness to initiate mental health support for children are needed to identify causalities and relevant factors for attitude changes and to draw reliable conclusions for future prevention activities.

Nevertheless, in line with the representative results from our analysis in Germany, several conclusions for prevention activities can be made. First, universal prevention activities have to be initiated or reinforced. The whole public population needs to be addressed, with low-threshold information being made available regarding the causes and influencing factors regarding mental disorders, how to detect them in young people, treatment and prevention options and how to offer support to children in need. People need to be made aware that children are entitled to professional support for mental health problems, just as they are for medical care when suffering from physical health problems. The existing literature shows that once mental health literacy is manifested, stigma decreases [73,74], indicating that facts help to overcome stigmata. In particular, to address the stigmatizing attitudes of formal and informal bystanders, high-profile information with facts about mental illnesses, the benefits of treatment and the treatability of mental disorders is essential and must be pushed forward [50,62]. Channels such as social media, advertising and websites [70,72] or material such as information flyers, posters or short films in public spots and institutions such as primary care can help with dissemination and raising awareness [73]. As health literacy appears to be a substantial factor for better mental health literacy in the empirical literature [38], improving health literacy in the same ways seems to be beneficial.

Furthermore, contact with affected people has been shown to reduce stigma [75,76]. Therefore, one implication to address stigmatizing attitudes in the German population should be to foster projects where people with mental disorders participate and speak about their experiences as well as disseminate knowledge. Universities, offices and schools can be possible locations and target groups [73].

In addition, in the area of the health care system, consideration needs to be given to external incentives for mental health prevention measures. In Germany, for example, you receive bonus points for regular check-up visits at the dentist, so if you need dentures at some point, the health insurance will grant a higher subsidy to the costs. To conclude, “bonus points” for mental health prevention could be taken into consideration for policy makers to promote population-based preventive mental health care.

At a more selective and indicated prevention level, which means when children are referred to mental health services, clinicians can directly support a reduction in stigma. They can assist the person (regardless of if it is a parent or a bystander) who initiated the help-seeking process for the child with regular consultations about experiencing stigma [18] and can support the initiator with finding appropriate and targeted help. This is also intended to create awareness and attention for this topic in the clinical context so that preventive measures can be taken. In this way a destigmatization and normalization of mental distress and help-seeking can be fostered, and strategies can be developed to counter negative attitudes in the social context of the child.

## 5. Conclusions

Our study showed that, in addition to parents, the social environment of a child is decisive in the initiation of mental health support for children in need. Nevertheless, it is evident that stigma is still widespread among the adult population at an average level. This raises concerns that mental distress in children is still often condemned rather than treated. An important approach to breaking out of this spiral is to initiate broad-based public information campaigns and to create opportunities to meet with affected persons. This could reduce the stigmatization that is often caused by a lack of knowledge about mental health and its treatment. Further, clinicians can assist the gatekeeper for the child’s service use with emotional support and targeted help when experiencing stigma. In addition, the initiation of incentives for acute and preventive mental health service use could be a further measure to reduce the high number of untreated children and adolescents and therefore promote public health.

## Figures and Tables

**Table 1:** Sociodemographic description of the main sample (all participants).

Characteristics of Participants in Main Sample (N = 1928)		
Gender	924 (47.93%) Male, 1003 (52.02%) Female, 1 (0.05%) Nonbinary	
Age	Min: 16; Max: 101; Mean: 55.36; Standard Deviation: 15.30	
Education	1 (0.05%)	not specified/other graduation
	5 (0.26%)	pupil of a general education school
	42 (2.18%)	without main school diploma
	577 (29.93%)	main school diploma
	603 (31.28%)	secondary school leaving certificate
	219 (11.36%)	graduation from polytechnic high school
	65 (3.37%)	specialized school degree
	224 (11.62%)	general/subject-linked university entrance qualification
	192 (9.96%)	completed university studies/studies at a university of applied sciences
Equivalent income	169 (8.77%)	< EUR 1000
	400 (20.75%)	EUR 1000–149,999
	523 (27.13%)	EUR 1500–199,999
	458 (23.76%)	EUR 2000–249,999
	378 (19.61%)	> EUR 2500
Adult history of psychotherapy	70 (3.63%)	Current psychotherapeutic/psychiatric treatment
	214 (11.10%)	Previous psychotherapeutic/psychiatric treatment
	1644 (85.27%)	Never had a psychotherapeutic/psychiatric treatment
Family status	2 (0.10%)	No answer
	723 (37.50%)	No children
	712 (36.93%)	Child(ren) and married/cohabitating
	491 (25.47%)	Child(ren) and single/divorced/widowed/married and living separately
Readiness to disclose mental health problems to social environment	9 (0.47%)	No answer
	202 (10.48%)	I totally disagree
	268 (13.90%)	I agree less
	624 (32.37%)	I partly agree
	474 (24.59%)	I pretty much agree
	351 (18.21%)	I fully agree
Readiness to disclose physical/somatic health problems to social environment	13 (0.67%)	No answer
	119 (6.17%)	I totally disagree
	235 (12.19%)	I agree less
	621 (32.21%)	I partly agree
	538 (27.90%)	I pretty much agree
	402 (20.85%)	I fully agree

**Table 3:** Results from the heteroscedastic ordered probit model. Notes: ^ƚ^ *p* < 0.1; * *p* < 0.05; ** *p* < 0.01; *** *p* < 0.001.

Dependent Variable	Adult Help-Seeking for Children	
**Independent Variables**	**Model 1**	**Model 2**
Stigmatizing attitudes	−0.05 *** (0.005)	−0.01 *** (0.003)
Gender		0.04 * (0.02)
Age		−0.001 * (0.001)
Education		0.004 (0.01)
Income		−0.3 × 10^−4^ ** (9.55 × 10^−6^)
Adult history of psychotherapy		0.04 ^ƚ^ (0.02)
Preventive health care		0.02 *** (0.005)
Family status		0.01 (0.01)
Readiness to disclose health problems		0.08 *** (0.02)
Cut 1	−1.92 (0.12)	−0.26 (0.07)
Cut 2	−1.58 (0.10)	−0.06 (0.07)
Cut 3	−1.00 (0.07)	0.25 (0.10)
Cut 4	−0.40 (0.05)	0.53 (0.14)
Log likelihood	−2524.19	−2256.69
LR Chi2	140.38 ***	522.74 ***
LR test on homogeneity	18.56 ***	89.79 ***
N	1906	1906

## Data Availability

Not applicable.
